# Adenosine deaminase activity in type 2 diabetes mellitus: does it have any role?

**DOI:** 10.1186/s12902-018-0284-9

**Published:** 2018-08-20

**Authors:** A. Niraula, S. Thapa, S. Kunwar, M. Lamsal, N. Baral, R. Maskey

**Affiliations:** 1 Department of Biochemistry, B.P. Koirala Institute of Health Sciences, Dharan, Nepal; 2 Department of Biochemistry, Kathmandu University School of Medical Sciences, Kavre, Dhulikhel, Nepal; 3 Department of Biochemistry, Modern Technical Institute, Satdobato, Lalitpur, Nepal; 4 Department of Internal Medicine, B.P. Koirala Institute of Health Sciences, Dharan, Nepal

**Keywords:** Adenosine deaminase, Diabetes mellitus type2, Hemoglobin A1c protein human, Blood glucose/metabolism

## Abstract

**Background:**

Diabetes mellitus is a group of metabolic disorders of carbohydrate metabolism in which glucose is underused, producing hyperglycemia. Diabetic patients are prone to opportunistic infection, thus serum ADA levels in these patients is very important as a screening test for Tuberculosis and autoimmune diseases. Thus, the present study was conducted to estimate the Serum ADA activity, glycated Haemoglobin (HbA1c), fasting and postprandial glucose level in patients with T2DM and to correlate the serum level of ADA with glycated Hemoglobin (HbA1c), fasting and postprandial glucose level in T2DM.

**Methods:**

This is a Hospital based cross-sectional study done in BPKIHs, Dharan, Nepal. 204 diagnosed patients (102 males and 102 females) with T2DM and 102 healthy controls were enrolled in the study. Diabetic patients were categorized into Uncontrolled and Controlled Diabetes on the basis of HbA1C; HbA1c > 7% = Uncontrolled Diabetes, HbA1c < 7% = Controlled Diabetes.

**Results:**

Serum ADA levels (U/L) was significantly raised in Uncontrolled Diabetic patients (49.24 ± 16.89) compared to controlled population (35.74 ± 16.78) and healthy controls (10.55 ± 2.20)**,** *p* value < 0.001. A significant positive correlation was obtained between Serum ADA and HbA1c, Fasting Plasma Glucose and Post-prandial Glucose respectively.

**Conclusion:**

There is a significant increase in Serum ADA activity in DM with increase in HbA1c levels which may play an important role in predicting the glycemic and immunological status in these patients.

## Background

Diabetes mellitus (DM) refers to a group of common metabolic disorders that share the phenotype of hyperglycemia [1]. There is an estimated 143 million people worldwide suffering from diabetes [2], almost five times more than the estimates ten years ago. This number may probably double by the year 2030 [3]. Data in Southeast Asia highlights that more than 436,000 people have been affected by Type 2 diabetes Mellitus (T2DM) and there is a definite probability for the number rising to 1,328,000 by 2030 [4]. The chronic hyperglycemia of diabetes is associated with long-term damage, dysfunction, and failure of various organs, especially the eyes, kidneys, nerves, heart and blood vessels. Autoimmune destruction of theβcells of the pancreas with consequent insulin deficiency and abnormalities that result in insulin resistance are the processes involved in the development of diabetes [5]. Also, T2DM has been always pigeon-holed as an intricate metabolic syndrome with multifactorial etiologies. The disease has been characterized by an atypical metabolism of all biomolecules i.e. carbohydrates, fats and proteins. Thus, collectively leading to an increased levels of blood glucose and lipid levels within the blood [6, 7].

Insulin resistance is associated with low-grade tissue specific inflammatory responses induced by various pro-inflammatory with oxidative stress mediators notably pro-inflammatory cytokines such as Interleukin-1 beta, Interleukin-6, Tumor Necrosis Factors- alpha along with numerous adipocytokines and chemokines, epigenetic factors and other transcriptional and metabolic pathways. Moreover, chronic exposure of pro-inflammatory mediators stimulates the activation of cytokine signaling proteins which ultimately block the activation of insulin signaling receptors in β-cells of pancreatic islets [7–9]. Adenosine deaminase (ADA) is a polymorphic enzyme that catalyzes the irreversible deamination of adenosine to inosine and has an important role in regulating adenosine concentration. Inosine and 2′-deoxyinosine are converted to the metabolic products i.e. hypoxanthine, xanthine and consequently to uric acid [10]. ADA is still considered as a marker for the assessment of cell mediated immunity [11]. ADA is suggested to be an important enzyme for modulating the bioactivity of insulin [12], but its clinical significance in T2DM has not yet been proven. In cases of oxidative stress and cell membrane damage, serum ADA is increased [13].

Previously, ADA has been reported to be a marker for insulin function [10], [13]. But, the correlation between ADA in diabetic patients has not been studied extensively in our country. Even though there are some reports available on ADA levels in diabetic subjects, these are all inconclusive and controversial. Since a relationship exists between ADA and cell mediated immunity [14]. This study was undertaken as a preliminary study to determine its serum activity and highlight its importance in the immuno-pathogenesis of T2DM. Thus, the present study was conducted to estimate the Serum ADA activity, glycated Haemoglobin (HbA1c), fasting and postprandial glucose level in patients with T2DM and to correlate the serum level of ADA with glycated Hemoglobin (HbA1c), fasting and postprandial glucose level in T2DM.

## Methods

### Study design

Hospital based comparative cross-sectional study conducted in the Department of Internal Medicine and Department of Biochemistry at B. P. Koirala Institute of Health Sciences (BPKIHS), Dharan.

### Sample size

Two hundred four patients diagnosed with T2DM. 102 healthy patients served as control.

### Sampling technique

Diabetic patient were selected from medicine OPD by convenient sampling technique and the biochemical parameters was assessed in Department of Biochemistry, BPKIHS. Control patients were recruited from routine laboratory (Department of Biochemistry).

### Inclusion criteria

Newly diagnosed and follow-up cases of T2DM visiting medicine OPD.

### Exclusion criteria

Those having any other chronic disease. Patients having any complications due to diabetes.Blood was collected in EDTA and serum vial from the study population for fasting and postprandial blood glucose. Serum was separated and stored at − 20 °C until test was performed. Obtained serum sample was used for the analysis of biochemical parameters. Serum ADA was performed by the manual method described by Giusti and Galanti, (1984) [15]. The unit for ADA is expressed in Enzyme units/litre (U/L).

Sodium hypochlorite and phenol Enzyme cascade Adenosine + H_2_O → Ammonia + Inosine

Alkaline Medium Ammonia+ Phenol+ Hypochlorite → Blue Indophenol Complex.

HbA1c was done by turbidimetric inhibition immunoassay (TINIA) method in Cobas c311 Autoanalyser [16]. HbA1c is expressed in percentage (%). The normal range is 4.5–6.3%.

The HbA1c determination is based on the turbidimetric inhibition immunoassay (TINIA) for hemolyzed whole blood.

- Glycohemoglobin (HbA1c) in the sample reacts with anti-HbA1c antibody to form soluble antigen-antibody complexes. Only one specific HbA1c antibody site is present on the HbA1c molecule, complex formation does not take place.
- Addition of R2 (Polyhapten reagent) and start of reaction: The polyhaptens react with excess anti-HbA1c antibodies to form an insoluble antibody-polyhapten complex measured turbidimetrically

Fasting blood glucose (FPG) and Postprandial blood glucose (PPG) was done by Hexokinase Method (Cobas c311 Autoanalyser). FPG and PPG is expressed in mg/dl. DM was classified as Controlled and Uncontrolled on the basis of HbA1c level (< 7% = Controlled & > 7% = Uncontrolled) respectively. Data was collected and entered using Microsoft Excel™ and analyzed using Statistical Package of Social science (SPSS) version 11.5. Data was expressed in terms of figure, percentage, mean and standard deviation. Independent sample t-test was used to compare the baseline and biochemical data between controlled and patients with T2DM. Analysis of variance (ANOVA) was used to compare the ADA level between healthy controls and controlled and uncontrolled T2DM. Spearman’s correlation was applied to correlate serum ADA level with different markers for glycemic control viz., HbA1c, FPG and PPG respectively.

Ethical approval was taken as per the guidelines by Institutional Review Committee (IRC, BPKIHS) on October, 2015 (IRC No: 568/072/073-IRC).

## Results

The study population comprised of total of 204 participants diagnosed of T2DM and 102 healthy controls. Demographic data has been depicted in Table 1**.** Biochemical parameters showed that Mean serum ADA was significantly higher in Diabetic patients compared to healthy controls as illustrated in Table 2. Further, ADA levels was significantly higher in Uncontrolled T2DM than in controlled T2DM as shown in Table 3 respectively. Spearman’s Correlation of FPG, PPG, and HbA1c with serum ADA showed a significant positive correlation with all of the three glycemic parameters. This suggests the use of ADA as an alternative marker of Glycemic control in Diabetic patients. Serum ADA showed a significant positive correlation with HbA1c which is considered as a good marker for long term glycemic control. Hence, it highlights the role of ADA as a potential marker for glycemic control as depicted in Table 4**.**

**Table 1:** Baseline characteristics of the study population

Characteristics	Type 2 DM	Control	*p* value
Gender (Male/Female)	103/101	51/51	–
Age	54.82±12.16	45.5±10.4	0.01^a*^
Systolic Blood Pressure (SBP) (mm Hg)	130±4.5	110±5.6	0.002 ^a*^
Diastolic Blood Pressure (DBP) (mm Hg)	95±5.8	80±4.5	0.04 ^a*^
BMI (kg/m^2^)	26.12±1.8	22.4±2.5	0.02 ^a*^

^a^Independent sample t-test

*p-value < 0.05 is considered to be statistically significant

**Table 2:** Comparison of biochemical parameters in the study population

Biochemical Parameters	Type 2 DM	Control	*p*-value
Fasting Plasma Glucose (FPG) (mg/dL)	153.64±85.84	90.24±25.54	0.001^a*^
Postprandial Glucose (PPG) (mg/dL)	242.72±136.63	130.54±35.55	0.001^a*^
HbA1c (%)	6.78±2.48	3.4±0.55	0.001^a*^
Serum ADA (Units/Litre)	40.44±17.97	10.55±2.20	0.001^a*^

a = Independent sample t-test

*p-value < 0.05 is considered to be statistically significant

**Table 3:** Mean ADA level in healthy control, controlled and uncontrolled T2DM

Variables	Serum ADA	*p*-value
Control	10.55±2.20	< 0.001^a^*
Controlled Diabetes Mellitus (*n*=133)	35.74±16.78	
Uncontrolled Diabetes Mellitus (*n*=71)	49.24±16.89	

^a^ANOVA

*p-value < 0.05 is considered to be statistically significant

**Table 4:** Spearman’s rho correlation correlating the level of ADA with FPG, PPG and Hba1c in T2DM

Parameters	ADA	FPG	PPG	HbA1c
ADA	-	0.33	0.306	0.417
		< 0.001	< 0.001	< 0.001
FPG	0.333	-	0.742	0.73
	< 0.001		< 0.001	< 0.001
PPG	0.306	0.742	-	0.691
	< 0.001	< 0.001		< 0.001
HbA1c	0.417	0.733	0.691	-
	< 0.001	< 0.001	< 0.001	

*p-value < 0.05 is considered to be statistically significant

## Discussion

T2DM is the predominant form of diabetes worldwide, accounting for 90% of cases globally [17]. It is a multifactorial systemic disease with hereditary and environmental causes being the major attributable factor. These lead to insulin resistance and defective secretion of insulin by pancreatic beta-cell. Immunological disturbances of cell-mediated origin are believed to initiate from T-lymphocyte dysfunction. Invitro studies have shown that in T2DM, inappropriate immune responses may result from the defects in the action of insulin that is required for the function of T-lymphocytes [18]. ADA plays a crucial role in lymphocyte proliferation and differentiation and shows its highest activity in T- lymphocytes [17, 18]. The present study shows a significant elevation in the ADA levels (U/L) in uncontrolled diabetic subjects (49.24 ± 16.89) compared to controlled population (35.74 ± 16.78). The high plasma ADA activity might be due to abnormal T-lymphocyte responses or proliferation; may point towards a mechanism that involves its release into the circulation [19, 20]. Hence, we suggest that increased ADA activity in diabetic individuals could be due to altered insulin related T-lymphocyte function or due to increased immunological dysfunction. Previously, Chang and Shaio, have demonstrated that impaired cell mediated immunity was associated with abnormal lymphocyte proliferation [21].

This study shows that ADA is raised in patients with diabetes mellitus, with values increasing in uncontrolled compared to controlled T2DM. Thus, the altered serum ADA levels may help in predicting immunological dysfunction in diabetic individuals. The present study depicts that there is a significant positive correlation between Serum ADA levels and Hba1c, which suggests that Serum ADA level can also be used as a biomarker in predicting glycemic control in diabetic patients. Studies have shown that there is a direct correlation with the expression and activity of ADA with the extent of severity of inflammation, as T2DM is associated with chronic hyperglycemia and an ongoing low-grade systemic inflammation [22]. The role of ADA in the cellular immunity was first identified in patients with severe combined immune-deficiency (SCID) [23, 24]. The high activity of this enzyme was considered to be a reflection of immunological disturbances observed in tuberculosis [25] infectious mononucleosis [26], jaundice [27], leukemia [28] and other conditions ( [29–31]).

## Conclusion

Assessment of Serum ADA level is cost-effective and the efficient use of this biomarker may help in establishing this enzyme as a good marker for assessing cell-mediated immunity in diabetic individuals. Thus, we conclude that elevated ADA activity may be an important indicator in the immuno-pathogenesis of T2DM and can also be implicated as a biomarker for predicting glycemic control in diabetic individuals.

## Abbreviations

DM: Diabetes Mellitus
FPG: Fasting plasma glucose
HbA1c: Glycated Hemoglobin
PPG: Post-prandial plasma glucose
T2DM: Type 2 Diabetes Mellitus
